# Advancing Epidermal Growth Factor Receptor (EGFR) Exon 20 Insertion-Mutated Non-small Cell Lung Cancer (NSCLC) Management Through Molecular Diagnostics and Targeted Therapies

**DOI:** 10.7759/cureus.82573

**Published:** 2025-04-19

**Authors:** Selvaraj Giridharan, Jawaher Ansari, Imran Hussain

**Affiliations:** 1 Oncology, Tawam Hospital, Al Ain, ARE; 2 Respiratory Medicine, University Hospital North Midlands, Stoke-on-Trent, GBR

**Keywords:** egfr mutation, exon 20 insertion, non-small cell lung cancer (nsclc), precision oncology, resistance mechanisms, targeted therapies

## Abstract

Non-small cell lung cancer (NSCLC) associated with epidermal growth factor receptor (EGFR) mutations has experienced notable therapeutic advancements; however, exon 20 insertion mutations continue to pose a significant challenge, demonstrating resistance to conventional EGFR inhibitors. Recent progress in molecular diagnostics and targeted therapies has introduced new research opportunities for NSCLC with EGFR exon 20 insertion mutations. Randomized controlled trials have shown that therapies, such as amivantamab, exhibit substantial efficacy, with combination strategies offering even greater potential. These advancements are underpinned by advanced diagnostic techniques, including next-generation sequencing and liquid biopsy, which enable precise mutation detection and real-time treatment monitoring. Nonetheless, challenges remain, including the management of toxicity, equitable access to therapies, and the need for comprehensive real-world data. Emerging therapies and innovative trial designs suggest a promising future for the management of NSCLC with EGFR exon 20 insertion mutations. This editorial examines how clinical trials and molecular diagnostics are advancing the management of EGFR exon 20 insertion-mutated NSCLC.

## Editorial

Background

The recognition of epidermal growth factor receptor (EGFR) mutations as oncogenic drivers in non-small cell lung cancer (NSCLC) has revolutionized the lung cancer treatment landscape over the past two decades. Common mutations, such as exon 19 deletions and L858R, respond robustly to tyrosine kinase inhibitors (TKIs), but exon 20 insertions, accounting for 4-12% of EGFR alterations, resist these agents due to structural changes in the kinase domain [1,2]. Historically, this resistance has confined patients to platinum-based chemotherapy, a suboptimal option. Recent innovations in targeted therapies and diagnostics, however, mark a turning point. This editorial examines how molecular diagnostics and targeted therapies are advancing EGFR exon 20 insertion-mutated NSCLC management, providing hope for a long-underserved patient population.

Historical context and therapeutic limitations

The treatment of NSCLC with EGFR exon 20 insertions has long been constrained by the limitations of conventional therapies. Platinum-based chemotherapy, the mainstay of care for decades, offers response rates of approximately 20-30% and a median progression-free survival (PFS) of 4-6 months [3]. However, its efficacy is tempered by its significant toxicity and lack of durable benefits. This approach, while standard, offered little in terms of long-term control, leaving patients with limited hope. Early attempts to extend TKI success to this subset faltered; agents such as erlotinib achieved response rates below 10%, with median PFS rarely exceeding three months due to steric hindrance in the mutated kinase domain [4]. This therapeutic gap persists despite advances elsewhere in NSCLC, highlighting a critical unmet need for strategies tailored to the molecular complexity of exon 20 insertions.

Recent therapeutic advances

The therapeutic landscape has undergone significant evolution with the advent of amivantamab, a bispecific antibody targeting both EGFR and MET. Unlike conventional TKIs, amivantamab addresses resistance mechanisms such as MET amplification, representing a substantial advancement [5]. This agent signifies a notable shift from traditional TKIs by employing dual-pathway inhibition to surmount resistance mechanisms. Subsequently, mobocertinib, a TKI specifically designed for exon 20 insertions, demonstrated a median PFS of 7.3 months in a phase 2 trial. However, a subsequent phase 3 study revealed its limitations, as it failed to surpass chemotherapy in the first-line setting, leading to its withdrawal from certain markets [6]. These findings highlight both the potential and limitations inherent in current targeted therapies. Further advancement is the exploration of combination regimens. The phase 3 PAPILLON trial evaluated amivantamab combined with carboplatin and pemetrexed and reported a median PFS of 11.4 months compared with 6.7 months with chemotherapy alone [7]. This combination leveraged amivantamab’s targeted inhibition of EGFR exon 20 insertions with the broad cytotoxic effects of chemotherapy to enhance tumor control, suggesting that integrating targeted agents with cytotoxic therapies improves efficacy. Collectively, these developments mark a shift toward more effective management of EGFR exon 20 insertion-mutated NSCLC driven by an improved understanding of its molecular underpinnings.

The role of molecular diagnostics

Central to these advances is the role of next-generation sequencing (NGS), which is indispensable in addressing the heterogeneity of over 100 documented exon 20 insertion variants [8]. Unlike polymerase chain reaction assays, which excel with common EGFR mutations, NGS identifies rare insertions and co-alterations, such as TP53 mutations or MET amplification, that shape prognosis and guide therapeutic selection [9]. For example, TP53 co-mutations often indicate a more aggressive disease course, necessitating tailored approaches. Liquid biopsy, which analyzes circulating tumor DNA, enhances this capability by offering a non-invasive means to monitor treatment response and detect resistance in real time, a capability that is increasingly vital as therapies evolve [10]. These diagnostics directly support targeted therapies; for instance, NGS-based patient selection in the CHRYSALIS trial enabled amivantamab to achieve a 40% objective response rate [5]. Together, these tools form the backbone of personalized care, ensuring that treatments align with each patient’s unique molecular profile.

Challenges in clinical implementation

Despite these strides, significant barriers have hindered their widespread adoption. Toxicity remains a pressing concern; amivantamab triggers infusion-related reactions (e.g., chills, fever, nausea, or dyspnea) in up to 66% of patients, requiring premedication and careful oversight, whereas the gastrointestinal effects of mobocertinib demand dose adjustments [5,6]. Multidisciplinary teams, oncologists, nurses, and pharmacists, are vital to manage these effects and optimize tolerability. Limited real-world data hinders long-term planning and sequencing strategies, while the high costs of NGS and novel therapies restrict access, particularly in underserved regions, deepening global disparities [11]. Policy reforms, such as expanded reimbursement and enhanced clinical infrastructure, are imperative to address these barriers. Next-generation TKIs such as furmonertinib and zipalertinib, under evaluation in trials like FURVENT and REZILIENT3, aim to boost potency and minimize off-target effects across diverse variants [12,13]. Combination strategies targeting the EGFR and MET pathways aim to preempt resistance and address common escape routes such as MET amplification [14]. Immunotherapy, although historically less effective in EGFR-mutated NSCLC due to its low tumor mutational burden, is being re-examined in combination trials, with early data suggesting possible synergy.

Future directions and research horizons**

Future advancements in EGFR exon 20 insertion-mutated NSCLC management will depend on deepening the role of molecular diagnostics [15]. Expanding NGS to routinely detect co-occurring mutations could further personalize treatment. Developing more sensitive liquid biopsy techniques will improve the early detection of resistance and enable proactive therapeutic shifts. Integrating artificial intelligence to analyze complex molecular data from NGS and liquid biopsies can enhance treatment response predictions. Trials such as LungMAP, which employs biomarker-driven therapy matching, should be expanded to accelerate these efforts. These strategies aim to shift the paradigm from palliative to sustained disease control.

Conclusion

The trajectory of EGFR exon 20 insertion-mutated NSCLC management reflects a broader evolution of precision oncology. From a condition marked by therapeutic challenge, it has transitioned to one with viable targeted options driven by advances in molecular understanding and drug development. However, realizing the full potential of these innovations requires a multi-faceted approach. Clinicians must navigate toxicity and evidence gaps; policymakers must address access disparities; and researchers must pursue novel therapies and diagnostics. Through sustained collaboration and commitment to evidence-based practice, the field can transform EGFR exon 20 insertion-mutated NSCLC into a condition amenable to long-term control, offering hope to patients who have historically faced limited options.
